# Resistance of Wheat Accessions to the English Grain Aphid *Sitobion avenae*

**DOI:** 10.1371/journal.pone.0156158

**Published:** 2016-06-01

**Authors:** Xiang-Shun Hu, Ying-Jie Liu, Yu-Han Wang, Zhe Wang, Xin-lin Yu, Bo Wang, Gai-Sheng Zhang, Xiao-Feng Liu, Zu-Qing Hu, Hui-Yan Zhao, Tong-Xian Liu

**Affiliations:** 1 State Key Laboratory for Crop Stress Biology in Arid Areas, Northwest A&F University, Yangling, Shaanxi, 712100, China; 2 Key Laboratory of Crop Pest Management on the Northwest Loess Plateau, Ministry of Agriculture, Northwest A&F University, Yangling, Shaanxi, 712100, China; 3 College of Agriculture, Northwest A&F University, Yangling, Shaanxi, 712100, China; CSIRO, AUSTRALIA

## Abstract

The English grain aphid, *Sitobion avenae*, is a major pest species of wheat crops; however, certain varieties may have stronger resistance to infestation than others. Here, we investigated 3 classical resistance mechanisms (antixenosis, antibiosis, and tolerance) by 14 wheat varieties/lines to *S*. *avenae* under laboratory and field conditions. Under laboratory conditions, alatae given the choice between 2 wheat varieties, strongly discriminated against certain varieties. Specifically, the ‘Amigo’ variety had the lowest palatability to *S*. *avenae* alatae of all varieties. ‘Tm’ (*Triticum monococcum*), ‘Astron,’ ‘Xanthus,’ ‘Ww2730,’ and ‘Batis’ varieties also had lower palatability than other varieties. Thus, these accessions may use antibiosis as the resistant mechanism. In contrast, under field conditions, there were no significant differences in the number of alatae detected on the 14 wheat varieties. One synthetic line (98-10-30, a cross between of *Triticum aestivum* (var. Chris) and *Triticum turgidum* (var. durum) hybridization) had low aphid numbers but high yield loss, indicating that it has high antibiosis, but poor tolerance. In comparison, ‘Amigo,’ ‘Xiaoyan22,’ and some ‘186Tm’ samples had high aphid numbers but low yield loss rates, indicating they have low antibiosis, but good tolerance. Aphid population size and wheat yield loss rates greatly varied in different fields and years for ‘98-10-35,’ ‘Xiaoyan22,’ ‘Tp,’ ‘Tam200,’ ‘PI high,’ and other ‘186Tm’ samples, which were hybrid offspring of *T*. *aestivum* and wheat related species. Thus, these germplasm should be considered for use in future studies. Overall, *S*. *avenae* is best adapted to ‘Xinong1376,’ because it was the most palatable variety, with the greatest yield loss rates of all 14 wheat varieties. However, individual varieties/lines influenced aphid populations differently in different years. Therefore, we strongly recommend a combination of laboratory and long-term field experiments in targeted planting regions to identify varieties/lines that consistently show high resistance to *S*. *avenae* infestation.

## Introduction

Wheat (*Triticum aestivum* L.) is a major grain crop worldwide. Cereal aphids cause serious wheat crop yield losses by reducing the number of grains and grain weight of wheat spikes. In addition, they are vectors of the *barley yellow dwarf virus* (BYDV), with the joint occurrence of the virus and aphids further increasing yield losses [1–2]. In China, about 23.4 million hectares of agricultural land is planted with wheat, of which 10–15 million hectares is infested with cereal aphids, resulting in 10% yield losses annually (Chinese National Agro-Tech Extension and Service Center [NATESC] database, http://www.natesc.moa.gov.cn/). The English grain aphid, *Sitobion avenae* (Fab.), is the dominant cereal aphid species, causing most damage at the wheat filling stage. This species is documented annually in the wheat fields of northern China, including the central part of Shaanxi Province [3].

Insecticide application is currently the main method used to control cereal aphids in China. However, this technique generates “3R” problems: residues, resistance, and resurgence [4], in addition to side effects on natural enemies during the early stages of wheat growth [5]. Several studies have documented lower *S*. *avenae* infestation on resistant wheat varieties. This phenomenon has been detected in England [6], the Czech Republic [7], Pakistan [8], France [9, 10], and Brazil [11]. The planting of resistant wheat cultivars represents an efficient and environmentally friendly strategy to prevent infestation, and has been successfully used in integrated pest management of the greenbug, *Schizaphis graminum* (Rodani), and the Russian wheat aphid, *Diuraphis noxia* (Kurdjumov) [12]. Even wheat varieties with low levels of resistance may substantially reduce aphid infestation. This is achieved by extending the developmental time of the aphids [13], which provides natural enemies with greater time to feed on them and prevent population explosions [14–16]. A financial return $600 is obtained by investing $1 in breeding resistant wheat varieties; however, only a return of $5 is obtained by the use of pesticides to control cereal aphids by farmers in the USA [17].

In the USA during the 1950s, hybridization techniques were used to breed a wheat variety that was resistant to the green bug; specifically ‘Amigo,’ which is a hybrid of wheat variety ‘Largo’ and rye (*Secale cereale* L.). Amigo has a single dominant resistant gene located on chromosome 7D, which originates from chromosome 1A of rye [18, 19]. In 1952, a population outbreak of the wheat aphid occurred in South Shanxi Province, China. Research showed that the wheat variety ‘Bima1’, which is a hybrid of wheat varieties ‘Biyumai’ and ‘Mazhamai,’ had better resistance to aphids and rust than other varieties. Consequently, ‘Bima’ wheat varieties were preferentially planted in China in the 1950s to control aphid damage [20].

The identification and evaluation of resistant wheat germplasm was initiated in the 1950–1960s in China [21–22]. A large number of wheat germplasms (including foreign germplasm and Wild Triticeae Dumort germplasm) resistant to *S*. *avenae* were identified over the subsequent 50 years [3, 23–44]. All directories of Chinese resistant wheat germplasms to *S*. *avenae* established over this timeframe are presented S1 Table. However, the mechanism and heredity of resistance in these germplasms has remained unclear. In parallel, major progress in developing transgenic resistant wheat to *S*. *avenae* was achieved in China [45–48]. However, most cultivated wheat varieties were identified as being aphid-susceptible in the Yellow and Huai Valley, which are the main wheat planting areas in China [38, 49].

The three classic resistance mechanisms of wheat crops to aphid pests are antixenosis (non-preference), antibiosis, and tolerance [50, 51]. Differences in aphid population levels may be attributed to differences in antixenosis and antibiosis (e.g., precocity), due to certain morphological and chemical characteristics of different wheat varieties. Tolerance is also an important resistance characteristic of wheat yield responses to the impact of aphid infestation. However, evaluation of wheat tolerance is costly, and time-consuming, with inconsistent result being produced from field experiments [23, 31, 49, 52].

Here, we conduct a comprehensive evaluation on the resistance mechanisms of 14 wheat varieties using the 3 classical resistance mechanisms. This research is expected to provide baseline information about the mechanisms that cause resistance in these varieties, and a theoretical basis on breeding resistant wheat varieties to reduce the impacts of aphids for integrated management of aphid.

## Materials and Methods

The experiments were performed in a climate chamber laboratory (25 ± 0.5°C (day) and 22 ± 0.5°C (night), with a 16 h light:8 h dark 114 photoperiod, and approximately 70 ± 10% relative humidity (R.H.)) and the experimental fields of Northwest A&F University (central Shaanxi Province, China; 34°17ʹ35ʺN latitude, 108°4ʹ18ʺE longitude).

### Wheat varieties (germplasms)

The 14 wheat varieties (lines) used in this study are listed in Table 1. Seven common wheat varieties were used. Specifically: (1) Batis, Astron, Ww2730, and Xanthus, obtained from Germany, (2) Amigo, and PI high, obtained from USA, (3) Xinong 1376, a native early-maturing cultivar, used as susceptible control [25, 26, 37]. The 6 synthetic wheat varieties/lines used were hybrid of *T*. *aestivum* and *T*. *monococcum* or *T*. *turgidum* (var. durum). An einkorn wheat line was used as a resistant control.

**Table 1:** List of wheat accessions.

Germplasm	Origin	Note
Xiaoyan 22	Hybrid of *T*. *aestivum* and *Thinopyrum ponticum*, susceptible control	Widely planted in northern China because of its high yield and stress tolerance
Xinong 1376 /Qianjinzao	*T*. *aestivum* from China	Early-maturing, susceptible to Chinese population
Tm	*T*. *monococcum*	Resistant to Chinese population
Amigo,	*T*. *aestivum*, from USA	with a 1AL1RS wheat-rye (*Secale cereale*) chromosome translocation [18, 19]
PI high	*T*. *aestivum*, from USA	High yield
Batis	*T*. *aestivum*, from Germany	Susceptible to German population [29]
Astron, Ww2730,	*T*. *aestivum*, from Germany	Resistant to German population [29]
Xanthus	*T*. *aestivum*, from Germany	
186Tm, Tam200, Tp	Hybrid of *T*. *aestivum* and *T*. *monococcum*	With resistant gene from *T*. *monococcum*
98-10-30, 98-10-35	Hybrid of *T*. *aestivum* (var. Chris) and *T*. *turgidum* (var. durum)	Resistant to German population [29]

### Aphids

In the wheat field population of Shaanxi Province, China, more than 95% aphids are *S*. *avenae*, and less than 5% are bird cherry-oat aphid *Rhopalosiphum padi* L. and greenbug aphid *Schizaphis graminum* (Rondani) [3]. Therefore, we used *S*. *avenae* aphids in our experiment.

The stock population of *S*. *avenae* aphids used in the antixenosis experiment was originally obtained from a single wingless individual that was found on a winter wheat plant in the experimental fields. The stock population was maintained in a separate cage on wheat seedlings (variety ‘Xiaoyan22’) in a climate chamber.

### Antixenosis experiments

Antixenosis represents a clear non-preference of a plant to serve as a host to an arthropod. Specifically, 3 experiments were conducted to determine the antixenosis of wheat varieties to *S*. *avenae* alatae (winged individuals).

The first antixenosis field experiment was conducted from 2003 to 2006. The wheat varieties/lines were sown on October of the previous year (2003 and 2005), and were harvested on June of the next year (2004 and 2006). Two fields were planted in 2003 and harvested in 2004, marked as 2004-A and 2004-B. A single, large field was planted in 2004 and harvested in 2005. This large field was divided into 3 sections, marked as 2005–1, 2005–2, and 2005–3. One field was planted in 2005 and harvested in 2006, and was marked as 2006. Over the 3-year period, each variety/line had a total of 6 replications in 6 fields/sections. Each replicated block of each variety/line covered a 20-m^2^ area. The blocks were 4 m long, and consisted of 20 rows with 25 cm spacing (total 5 m). Before planting, 10 kg/666.7 m^2^ of nitrogen (ammonium nitrate) and 25 kg phosphate fertilizer (potassium dihydrogen phosphate) were applied to the experimental plots. An additional top-dressing of 10 kg/666.7 m^2^ nitrogen was added at the stem elongation stage (March) of winter wheat. To avoid assessing the mutual effect between different wheat varieties/lines planted in neighboring plots, data were only collected from a 12 m^2^ (3 m × 4 m) area in the center of each repeated block. Natural infestation by adult *S*. *avenae* alatae was counted 3 times in the 12 m^2^ center area of each repeated block, with 1 week separation between the end of March to mid-April, This is because, during this period, alatae migrated from wheat fields in southern China [53, 54].

The second antixenosis experiment was performed under laboratory conditions from September 2011 to November 2011. In this experiment, aphid alatae were allowed to choose among the 14 wheat varieties. Five seeds from each variety/line were planted in separate plastic pots (i.e., 1 pot per variety). The 14 pots containing wheat seedlings at the 2-leaf stage were randomly arranged in a circle, and were covered with a 2 m × 2 m × 1.5 m rectangular gauze cage to stop the alatae from escaping. One-hundred adult aphid alatae were deprived of food for 24 h, and were then released into the center of the experimental plants in the cage. The number of adult aphid alatae that established on each variety/line was counted and recorded after 1, 2, 4, 8, and 24 h. This experiment was repeated 3 times. The randomly layout of pots within the three repeats was different.

The third antixenosis experiment was a palatability experiment, which was performed under laboratory conditions from February 2012 to May 2012. In this experiment, aphid alatae were allowed to choose between 2 wheat varieties. The 14 wheat varieties/lines were combined as 91 pairs (14 × 13 ÷ 2). The leaves of each paired combination of wheat leaves were cut to a length of 3 cm, and were placed on opposite sides of a 9-cm-diameter Petri dish. The bottom of the Petri dish was covered with filter paper, on which 2 ml distilled water was added to keep the leaves moist. Subsequently, 20 alatae aphids that had been deprived of food for 24 h were released in 2 batches onto the center of the Petri dish. After 1, 2, 4, 8, and 24 h, the number of aphids that had established on the leaves of each cultivar/line was counted and recorded. This experiment was repeated 3 times.

### Antibiosis experiments

Antibiosis is the antagonistic association between 2 species, in which at least 1 of the 2 species is adversely affected. It is a heritable quality possessed by a plant that adversely affects the life history or biology of the insect [50, 51]. In this study, the antibiosis experiments focused on population dynamics, and were performed between 2003 and 2006 in the same fields/sections that were used for the antixenosis experiments.

Data collection was performed in the 12 m^2^ central area of each repeated block. Five 1 m long rows were sampled per block. In this antibiosis experiment, all aphids (including alatae, apterae [wingless] adults, and nymphaea) from 5 points in each block were counted once a week from mid-March to the end of May (a total of about 10 times). Direct counts were used before the end of April, and scale counts, which were the average aphid number of 10 heads multiplied by count wheat head number in 1 m long rows, were used following the end of April. The maximum aphid number (MAN, or aphid peak number) and the aphid cumulative count (ACC) were integrated to elucidate aphid population development. The sum of the MAN and ACC at the 5 points in each repeat block was used as a single sample. The index of aphid susceptibility (IAS) was counted as:

IAS = the number of aphids at each point/ the average number of aphids at all points combined [2]

IAS ≤ 0.3 indicated high resistance, 0.3 < IAS ≤ 0.6 indicated intermediate resistance, 0.6 < IAS ≤ 0.9 indicated low resistance, 0.9 < IAS ≤ 1.2 indicated low susceptibility, 1.2 < IAS ≤ 1.5 indicated intermediate susceptibility, and IAS ≥ 1.5 indicated high susceptibility.

### Wheat tolerance experiment in the field

Tolerance is a genetic trait of a plant that enables it to tolerate higher pest populations than a susceptible cultivar before damage occurs [50, 51]. At plant maturity, the grain yield, the yield components, and their loss ratios were measured. The yield components included the number of heads in sampled 1 m rows, kernels per wheat head (the average of 30 heads), and the weight of 1,000 kernels (KW). The theoretical yield (TY) was calculated. The actual grain yields (AY) per 1 m row for all 5 sampled points were measured for each block. The TY, AY, and KW loss ratios were calculated using the following formula:

loss ratio = (control value - actual value)/ control value [2]

The control points were 1 m long rows. These points were located outside the 12-m^2^ core area, but within the 20-m^2^ area of each block. The control points were sprayed 3–5 times with imidacloprid (2.5% WP 8000X, 20 g a.i./hm^2^; Shijiazhuang Yaoyuan Pharmaceutical Technology Co., Ltd., Shijiazhuang, Hebei, China) at the start of April and end of May to eliminate all aphid infestation. When spraying, both sides of the control line were protected with cardboard to prevent the pesticide from reaching the other wheat plants [2, 3, 28, 29].

### Data analysis

There were 2 fields in 2004, 3 sections in 2005, and 1 field in 2006. The results for each field or section were assumed to be independent in different years; thus, the 6 fields/sections were treated as a single factor in the field experiments when using ANOVA. The number of adult aphid alatae was very small in early spring; thus, the total number of alatae obtained from each repeated block was combined as a single sample for each wheat variety/line. Then, two-way (14 wheat varieties and 6 fields/sections) ANOVA without duplication was used in the first antixenosis field experiment. The number of aphid alatae was analyzed using a repeatable one-way ANOVA in the second and third antixenosis laboratory experiments. MAN and ACC were transformed using natural logarithm transformation to ensure homogeneity of variance. The loss ratios of TY, AY, and KW were transformed using arcsine transformation to reduce variance. IAS were not transformed. Then, these parameters were analyzed using a two-way (14 wheat varieties and 6 fields/sections) ANOVA with the duplication of random factors model. All of the parameters were analyzed using ANOVA with General Linear Model (GLM). The means were separated by Tukey’s test under α = 0.05 (Data Processing System, DPS, software). All figures were drawn with SigmaPlot 12.1 (Systat Software Inc., Chicago, IL, USA).

## Results and Analysis

### Antixenosis experiment

**Fig 1:**
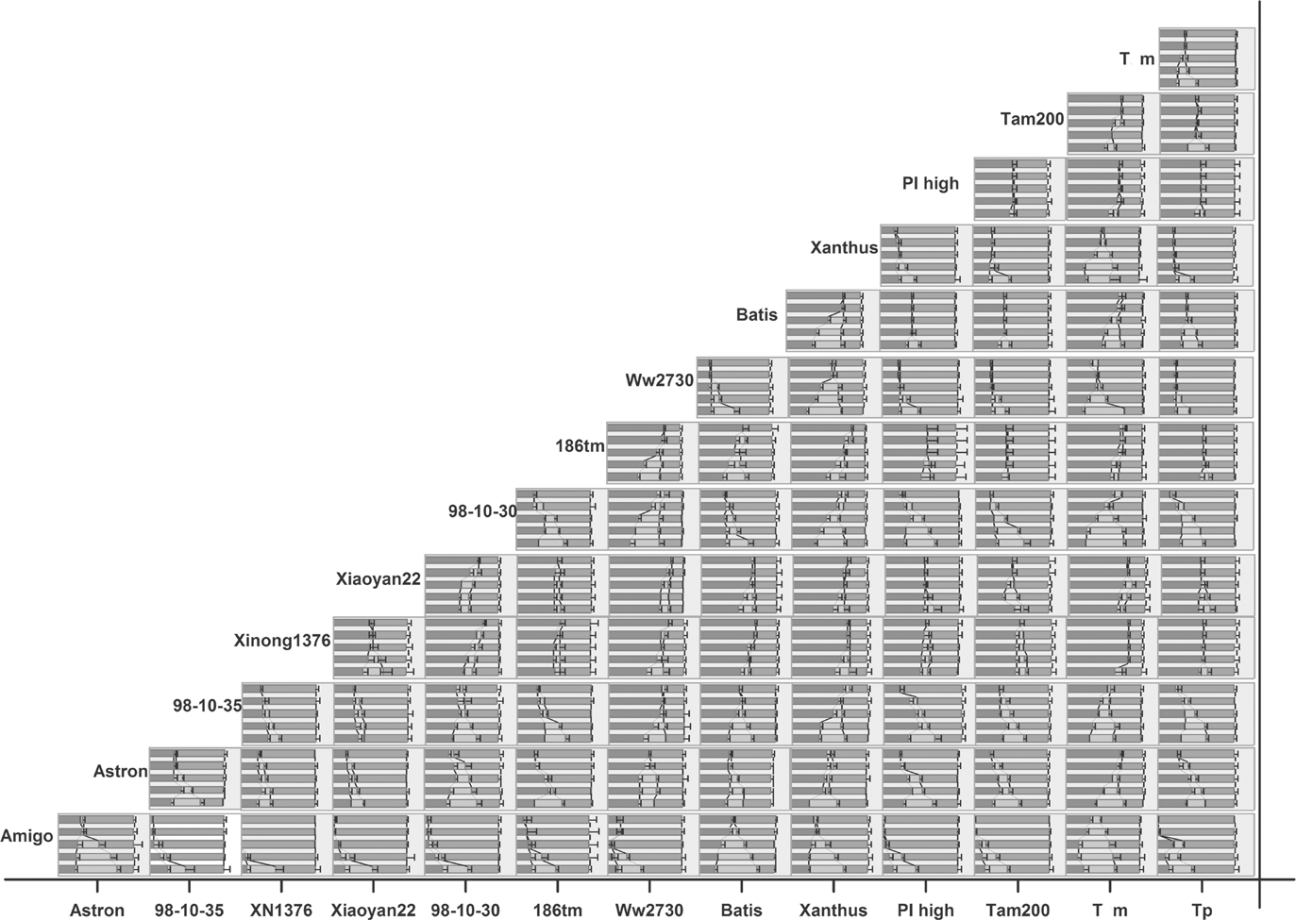
*S*. *avenae* alatae selection dynamics between 2 wheat varieties/lines. This figure includes 91 graphs (14 wheat varieties/lines) representing the random combination of 91 pairs. Each paired combination includes 2 different wheat varieties/lines. Each graph shows how 20 alatae selected between the 2 wheat varieties/lines (for each small graph of the paired combinations, the name of the variety on the left is on the horizontal axis, while the name of the variety on the right is above each graph) at 1 h, 2 h, 4 h, 8 h, and 24h (from the bottom up).

**Fig 2:**
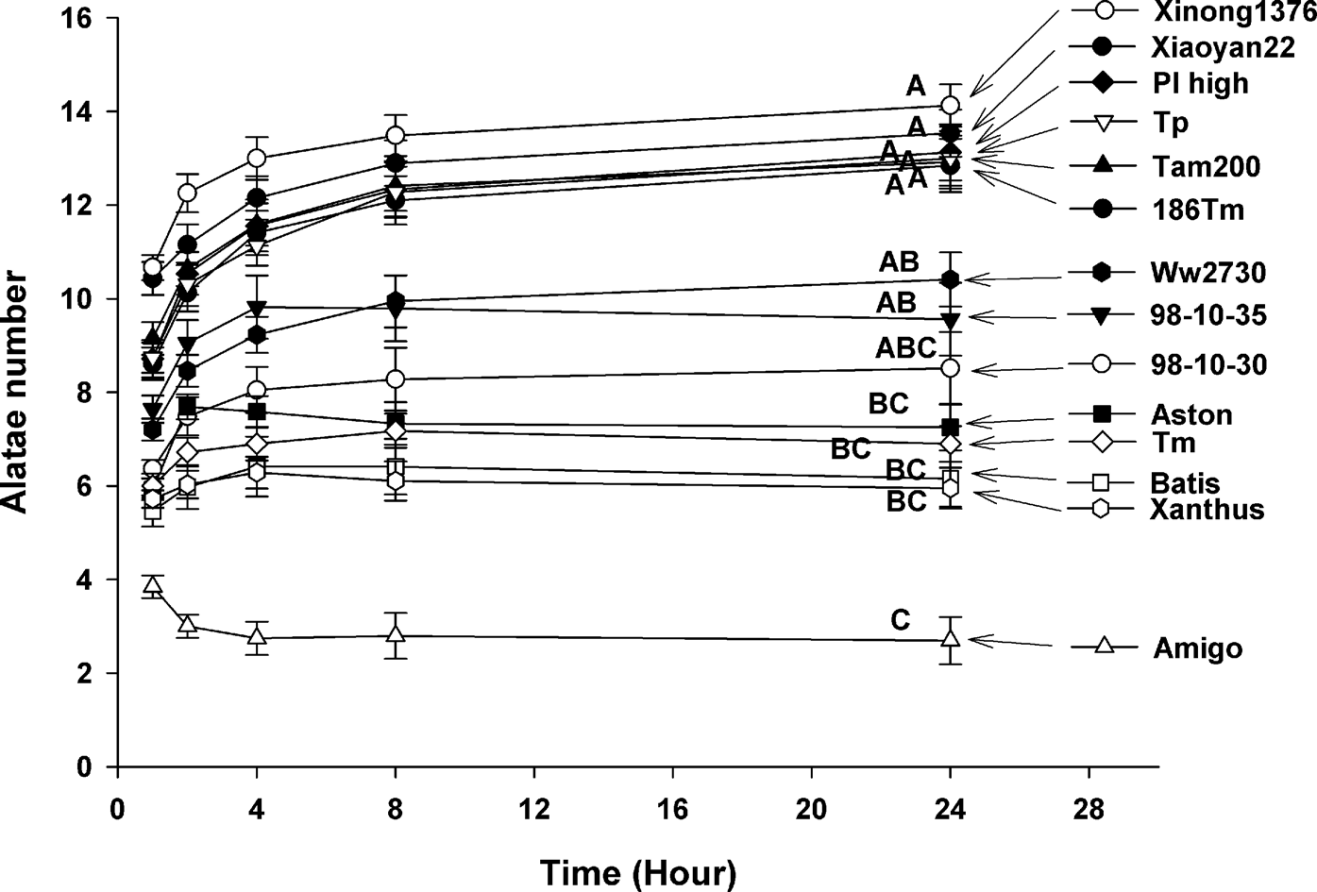
*S*. *avenae* alatae selection dynamics of the 14 wheat varieties at 1 h, 2 h, 4 h, 8 h, and 24 h (mean ± SD). Different capital letters indicate the significance of differences according to varieties after 24 h.

**Fig 3:**
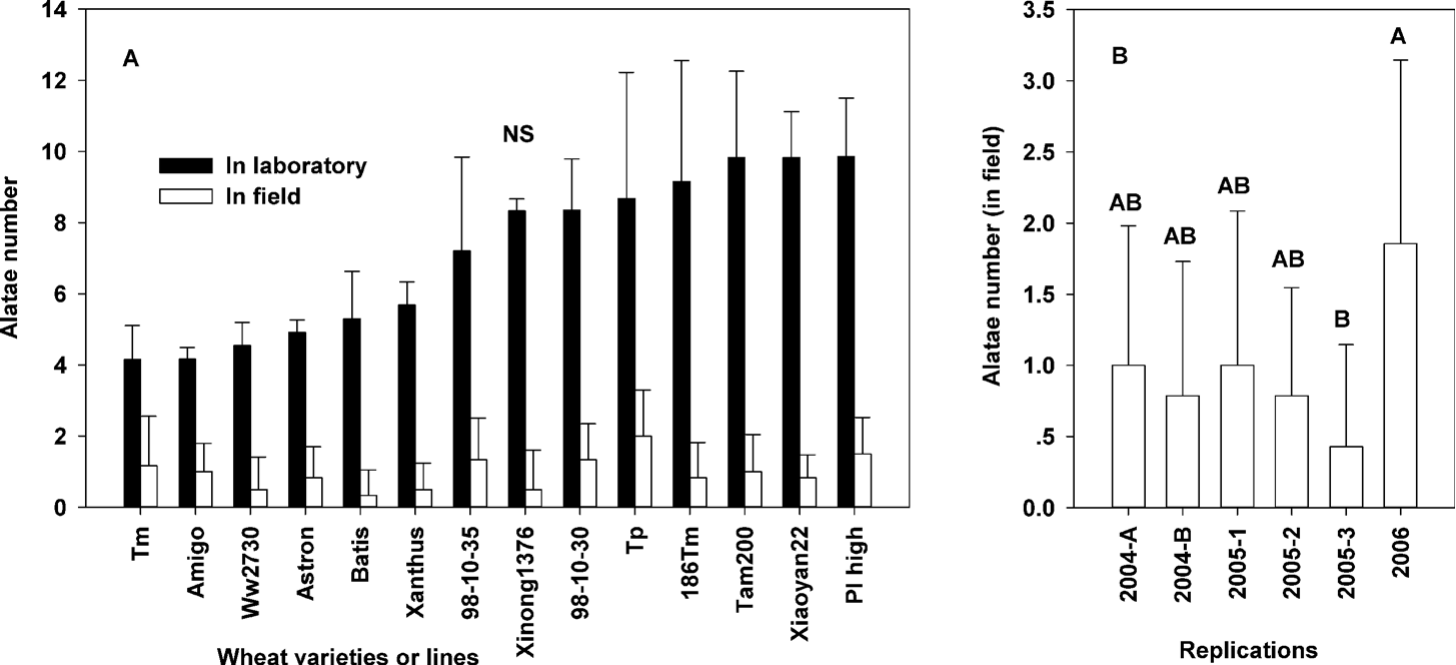
Average number of *S*. *avenae* alatae that chose each of the 14 wheat varieties/lines (mean ± SD). A: The alatae choose among 14 wheat varieties/lines in the laboratory after 24 h (black columns), and in the field during spring (white columns). B: Difference in the number of alatae among 6 fields/sections in 3 years. ‘NS’ above the bar in part A was not significant at *p* < 0.5. Different capital letters above the bar in part B indicate the significance of differences according to repeats.

In the palatability experiment (the first antixenosis experiment in the laboratory, where alatae chose between 2 wheat varieties/lines), significant differences in the average number of alatae on each variety/line were detected after 24 h (Figs 1 and 2) (*F* = 298; *D*.*F* = 13, 28; *p* < 0.001). After 8 h, aphid choosing behavior plateaued. After 24 h, the highest number of alatae was attracted to ‘Xinong1376,’ ‘Xiaoyan22,’ ‘PI high,’ ‘Tp,’ ‘Tam200,’ and ‘186Tm’ (from high to low), higher number of alatae was observed on ‘Ww2730,’ ‘98-10-35,’ and 98-10-30.’ The lowest number of alatae was observed on ‘Amigo,’ lower number of alatae attracted to each plant was observed on ‘Astron,’ ‘Tm,’ ‘Batis,’ and ‘Xanthus’ (from low to high) in 14 wheat varieties/lines (Fig 2). ANOVA indicated no significant difference in the percentage of adult aphid alatae attracted to each of the 14 wheat varieties/lines after 24 h in the non-preference experiment for the third antixenosis experiments in the laboratory (*F* = 1.49; *D*.*F*. = 13, 28; *p* = 0.18) (Fig 3A). The second antixenosis experiment also showed no significant different in the counts of adult aphid alatae attracted to each of the 14 wheat varieties/lines in field (*F* = 1.236; *D*.*F*. = 13, 83; *p* = 0.276). However, there were significant different between 6 fields/portions (*F* = 3.112, *D*.*F*. = 5, 83, *p* = 0.014) (Fig 3B). The total alatae was significantly greater in 2006 than that in 2005–3.

### Antibiosis field experiment

**Fig 4:**
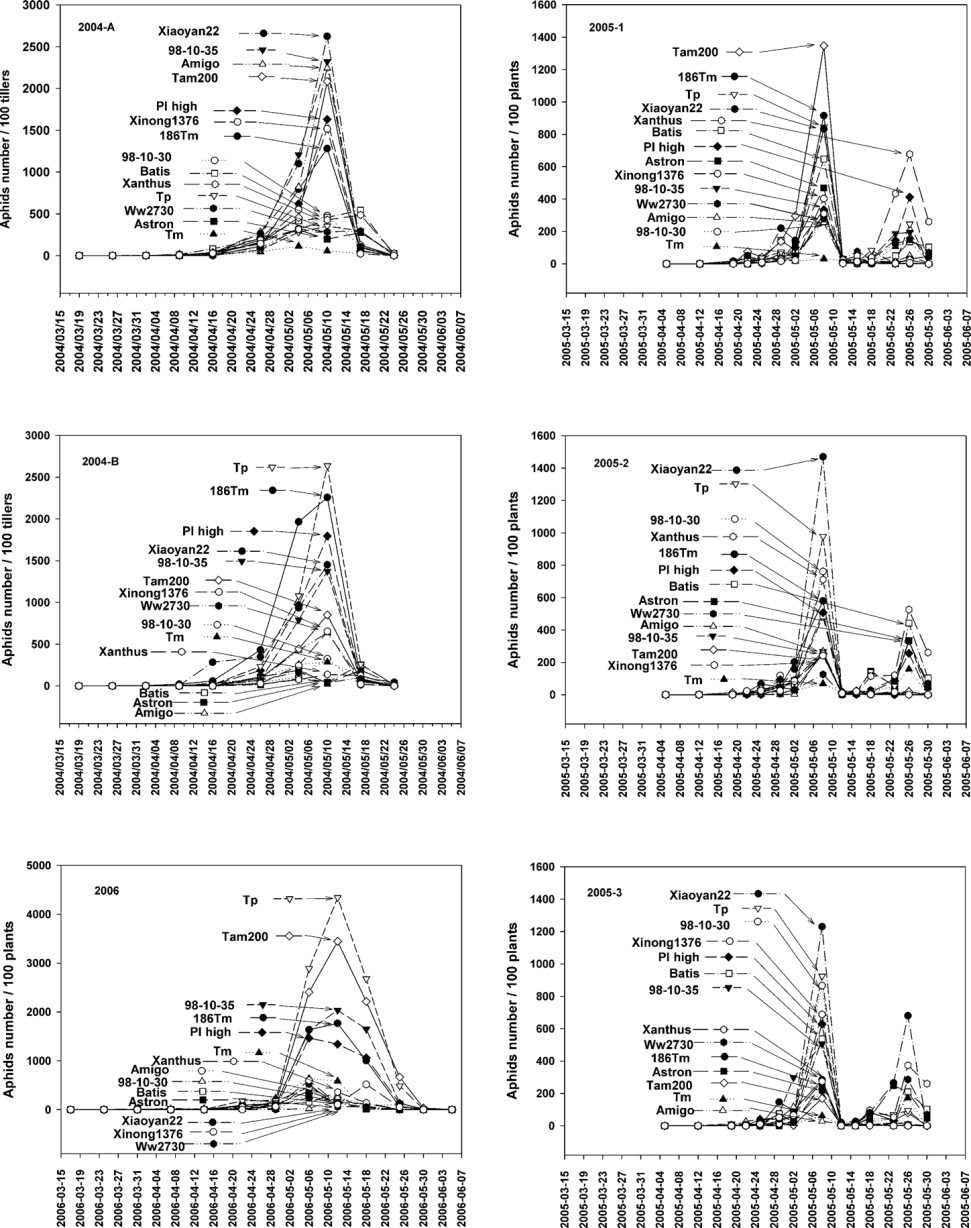
Population dynamics of *S*. *avenae* on 14 wheat varieties with 6 fields/sections over 3 years.

The abundance patterns of the *S*. *avenae* population were similar among all 14 wheat varieties/lines in all 6 fields/sections over the 3-year study period (Fig 4). Aphids were first detected on wheat seedlings in mid-March. Population numbers accelerated in mid-April. The MAN occurred in the first half of May each year. Population numbers declined in late May, and had disappeared by early June when the wheat was harvested.

**Fig 5:**
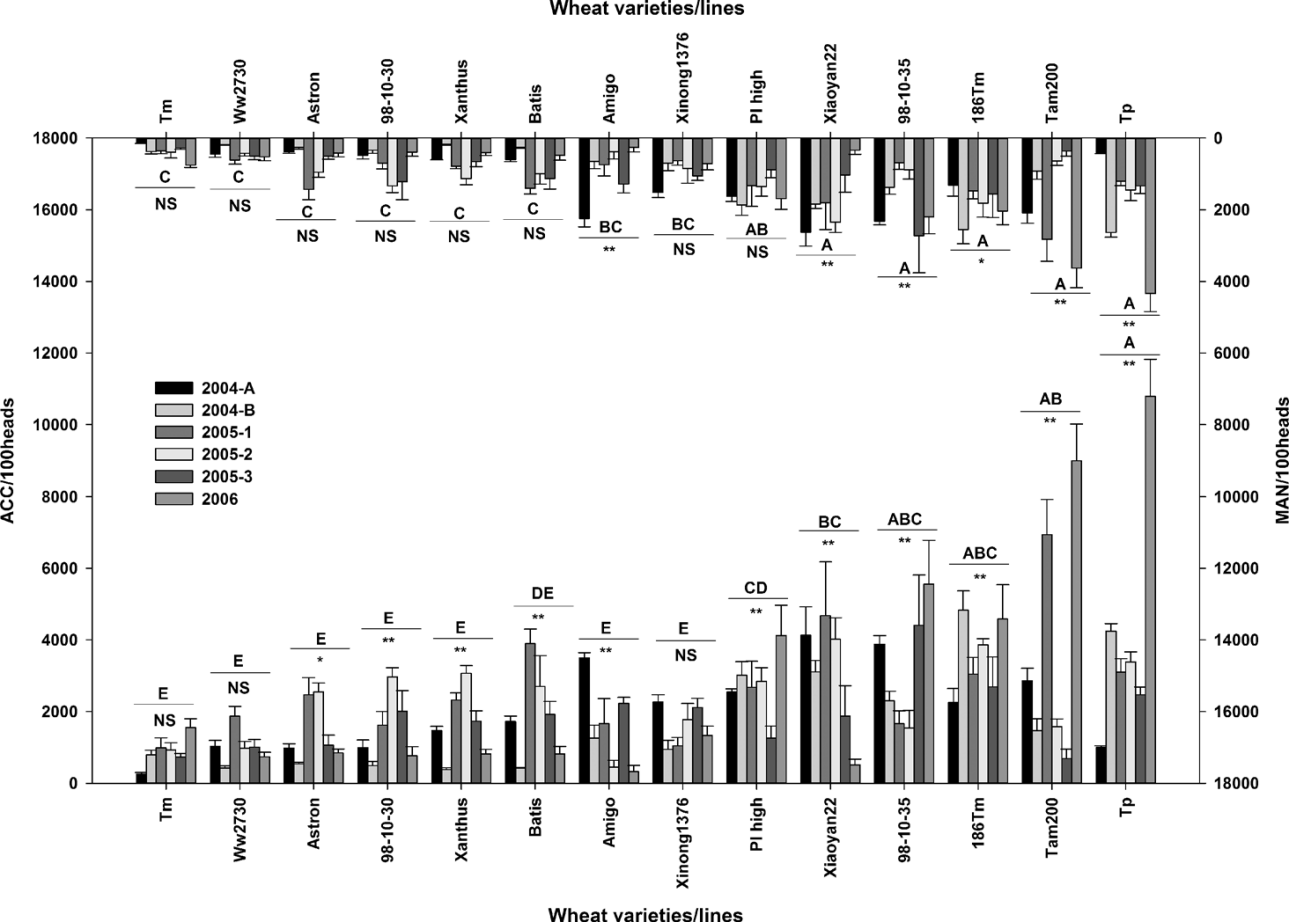
**The maximum aphid number (MAN, or aphid peak number, top x axes and right y axes) and the aphid cumulative count (ACC, below x axes and left y axes)for the 14 wheat varieties on 6 fields/sections over 3 years.** Different capital letters above the horizontal lines indicate the significance of differences of the average of the 6 fields/sections across the 14 wheat varieties/lines. Strongly significant (*p* < 0.01) values are marked as ‘**,’ significant (0.05 < p < 0.01) values are marked as ‘*,’ and non-significant (*p* > 0.05) values are marked as ‘NS,’ and are shown below the horizontal lines.

In the ANOVA-based random model with 2 factors (14 wheat varieties and 6 fields/sections) variation existed among the 14 wheat varieties/lines with respect to MAN and ACC (*F*_MAN_ = 3.47, *D*.*F*. = 13, 336, *p* = 0.0004; *F*_ACC_ = 2.76, *D*.*F*. = 13, 336, *p* = 0.0035). In contrast, ANOVA indicated no variation among the 6 fields/sections (*F*_MAN_ = 0.38, *D*.*F*. = 5, 336, *p* = 0.86; *F*_ACC_ = 1.37, *D*.*F*. = 5, 336, *p* = 0.25). Higher mean numbers of MAN and ACC were observed for ‘Tp,’ ‘Tam200,’ ‘186Tm,’ ‘98-10-35,’ ‘PI high,’ and ‘Xiaoyan22.’ These varieties/lines were considered to be susceptible to aphid infestation. Lower mean numbers of MAN and ACC were observed on ‘98-10-30,’ ‘Astron,’ ‘Xanthus,’ ‘Ww2730,’ and ‘Tm.’ These varieties/lines were considered to be relatively resistant to aphid infestation. There were significant interactions between the wheat varieties and replications (*F*_MAN_ = 6.12, *D*.*F*. = 65, 336, *p* < 0.001; *F*_ACC_ = 10.7, *D*.*F*. = 13, 336, *p* < 0.001). When checking the 14 wheat varieties, we found the MAN and ACC of 3 resistant (‘Tm,’ ‘Astron,’ and ‘Ww2730’) and 1 low susceptibility (‘Xinong1376’) lines/varieties were not significantly different among the 6 fields/sections (Fig 5).

The ANOVA based on the random model with 2 factors (14 wheat varieties and 6 fields/sections) showed a significant difference for IAS based on MAN and ACC among the 14 wheat varieties (*F*_IAS based on MAN_ = 5.45, *F*_IAS based on ACC_ = 8.97, *D*.*F*. = 13, 366, *p* < 0.0001) and interactions between the wheat varieties and replications (*F*_IAS based on MAN_ = 28.80, *F*_IAS based on ACC_ = 20.26, *D*.*F*. = 13, 366, *p* < 0.0001). There was no significant difference among the 6 fields/sections. The IAS identified ‘Tm’ and ‘Ww2730’ as stable resistant wheat germplasms in 6 fields/sections over the 3 years. ‘Xinong 1376’ was also identified as a stable wheat germplasm, but with low susceptibility over the same period. The IAS of ‘98-10-30,’ ‘Astron,’ ‘Xanthus,’ and ‘Batis’ was relatively stable, ranging between 0.5 and 1.2. The IAS of ‘Xiaoyan22,’ ‘Amigo,’ ‘Tam200,’ ‘Tp,’ ‘186Tm,’ ‘PI high,’ and ‘98-10-35’ fluctuated broadly across the 6 fields/sections over the 3 years.

### Wheat yield losses by the natural field aphid population (Tolerance)

**Fig 6:**
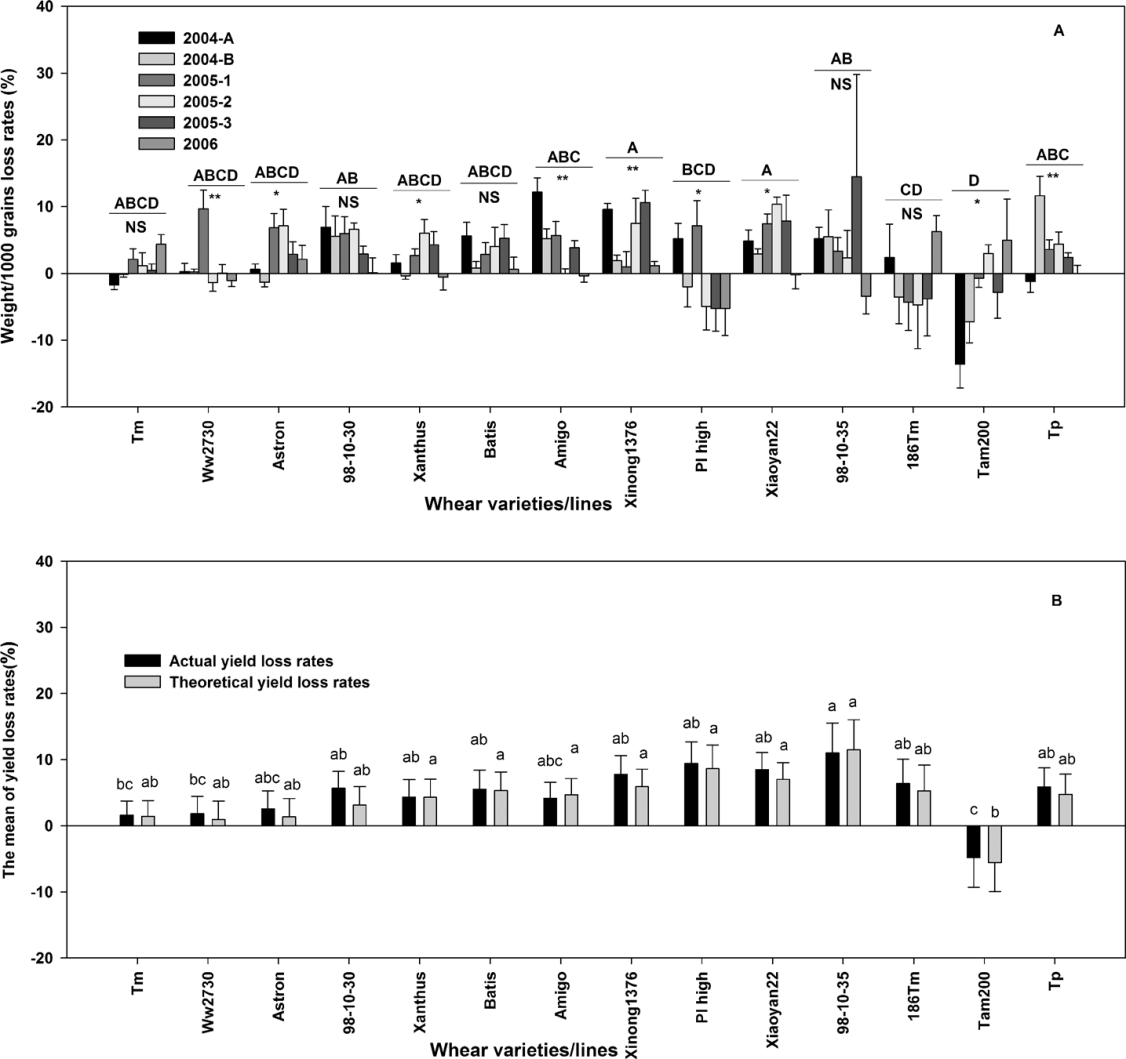
**The loss rates of the 1000 grain weight (KW), for the 14 wheat varieties on 6 fields/sections over 3 years (A), and the loss rates average of theoretical yield (TY) and actual yield (AY) for the 14 wheat varieties based 6 fields/sections over 3 years.** In part A, different capital letters above the horizontal lines indicate the significance of differences of the averages of the 6 fields/sections across the 14 wheat varieties/lines. Strongly significant (*p* < 0.01) values are marked as ‘**,’ significant (0.05 < *p* < 0.01) differences are marked as ‘*,’ and non-significant (*p* > 0.05) differences are marked as ‘NS,’ and are shown below the horizontal lines. In part B, different little letters above the bar indicate the significance of the differences for the averages of the varieties/lines (p < 0.05).

The ANOVA based on the random model with 2 factors (14 wheat varieties and 6 fields/sections) showed significantly different average loss rates for KW (*F* = 4.40, *D*.*F*. = 13, 366, *p* < 0.0001), TY (*F* = 3.89, *D*.*F*. = 13, 366, *p* < 0.0001), and AY (*F* = 4.27, *D*.*F* = 13, 336, *p* < 0.0001) among the 14 wheat varieties (Fig 6). In contrast, the ANOVA indicated no variation among the 6 fields/sections (*F*_KW_ = 2.15, *D*.*F*. = 5, 336, *p* = 0.06; *F*_TY_ = 0.70, *D*.*F*. = 5, 336, *p* = 0.63; *F*_AY_ = 1.77, *D*.*F*. = 5, 336, *p* = 0.12). The interactions between the wheat varieties and replications for KW (*F*_KW_ = 2.00, *D*.*F*. = 65, 336, *p* < 0.0001) was significant. However, a significant relationship was not detected for the interactions of the wheat varieties with TY and AY (*F*_TY_ = 0.99, *D*.*F*. = 65, 336, *p* = 0.51; *F*_AY_ = 1.10, *D*.*F*. = 65, 336, *p* = 0.29). The highest average KW, TY, and AY yield loss rates were obtained for ‘98-10-30,’ ‘Xinong1376,’ ‘PI high,’ and ‘98-10-35.’ The lowest average KW, TY, and AY yield loss rates were obtained for ‘Xanthus,’ ‘Amigo,’ ‘Astron,’ ‘Ww2730,’ and ‘Tm.’ Intermediate average KW, TY, and AY yield loss rates were obtained for ‘186Tm,’ ‘Tp,’ ‘Batis,’ and ‘Xiaoyan22.’

Highly variable average KW, TY, and AY yield loss rates were obtained for ‘Tam200,’ ‘Tp,’ ‘Xiaoyan22,’ ‘PI high,’ and ‘98-10-35.’ Gently fluctuating average yield KW, TY, and AY loss rates were obtained for ‘Xinong1376,’ ‘Astron,’ ‘Xanthus,’ ‘Batis,’ and ‘Amigo.’

## Discussion

Our study demonstrates major differences in the crop yield loss rates and IAS of the 14 wheat varieties/lines among repeats, fields, and years. Previous studies also detected great disparity among areas and years for crop yield loss rates and IAS of the same germplasm [2, 32, 43, 55–57]. These differences arise because *S*. *avenae* is not evenly distributed within fields, but they form aggregations [58–61], with generally weak spatial associations and some edge effects [62]. Consequently, wheat varieties that were identified as being resistant may not necessarily be so; rather, these varieties may have been outside the core aphid aggregation area within each field. Thus, the screening and identification of resistant wheat varieties should reflect these aspects in the field. However, several experimental repeats conducted on a long period of time are required to confirm our observations. Alternatively, artificially infesting fields might prevent aggregations forming, resulting in a more evenly distributed spread of aphids within fields.

In the UK, France, Denmark, Romania, and Chile [63–68], there is low genetic differentiation among *S*. *avenae* populations across different geographical regions. In contrast, *S*. *avenae* exhibits high genetic differentiation across different geographical regions in China, and may be divided into different biotypes [69–72]. For instance, most *S*. *avenae* that are found in the wheat fields of northern China originate from winged offspring that have migrated north from wheat fields in southern China [53, 54]. Some offspring also originate from eggs that overwinter locally or from adults in non-extreme cold years [73–75]. Consequently, aphids originate from multiple geographical populations in spring, resulting in high genetic differentiation. This issue may also cause the highly different resistance levels detected in crops across fields and years.

Our laboratory results show that there was a lack of consistency in aphid alatae attraction when given the choice between 14 wheat varieties versus 2 wheat varieties. Visual and olfactory discrimination is the first step used by insects to locate host plants. The grey glume, green head, and green stem of wheat plants have been reported to be significantly positively correlated with plant resistance to *S*. *avenae* [34, 76]. After the alatae land on the host leaf, gustatory discrimination is the next step, whereby individuals select the host plant based on taste. Our results showed that the alatae completed this process within 8 h. Differences in the level of selection between the 2 experiments may be because vision, olfaction and taste are used together when choosing among the 14 wheat varieties in field and a 2 m × 2 m × 1.5 m rectangular gauze cage, whereas taste is preferentially used when choosing between 2 wheat varieties in a 9-cm-diameter Petri dish.

Our MAN and ACC field results showed that ‘Amigo’ was the highest susceptible variety to *S*. *avenae* in 2004, but that it was the resistant in 2005 and 2006. Only 13.5% of alatae on average chose to feed on ‘Amigo’ when given the choice of 2 varieties. ‘Amigo’ contains a resistant gene to *S*. *graminum* biotypes B and C [18, 19]. ‘Amigo’ tends to be resistant to most aphid populations in different regions of China, and across different years [37, 70, 77]. However, this variety is susceptible in some geographic regions in some years in China [35, 70] and Germany [29, 77]. These results support the high genetic differentiation of *S*. *avenae* in China [69–72], and suggest Amigo has different resistance level to different biotypes or geographical population of *S*. *avenae*. Therefore, ‘Amigo’ could be used to distinguish the biotypes or geographical population of *S*. *avenae* in China [70].

We found that the average IAS of *S*. *avenae* on all wheat varieties/lines ranged from 0.4 to 0.8. Of note, fewer alatae chose the 4 German wheat varieties (‘Ww2730’, ‘Astron’, ‘Xanthus’, and ‘Batis’). Liu et al. reported that the IAS of *S*. *avenae* is about 1.0 on ‘Batis’ and ‘Ww2730’ at the adult plant stage in other fields in Yangling, Shaanxi Province [34]. In Germany, ‘Astron,’ ‘Xanthus,’ and ‘Ww2730’ were resistant to *S*. *avenae* populations, whereas ‘Batis’ was susceptible [28, 78]. ‘Astron,’ ‘Batis,’ and ‘Xanthus have a thick wax powder layer on the ear and leaf, while ‘Ww2730’ has thick tomentulose on the ear and leaf. In China, these 4 German varieties mature about 3 weeks later than Chinese wheat varieties. The electrical penetration graph (EPG) technique shows that *S*. *avenae* salivated more in the sieve elements of ‘Batis’ than ‘Ww2730,’ and spent more time passive sucking in the sieve elements of ‘Batis’ than ‘Ww2730,’ and ‘Ww2730’ also has other factors in the epidermis, mesophyll, and phloem that restrict feeding by aphids [79]. Thus, the resistant mechanisms of these 4 German varieties include antibiosis and non-preference to *S*. *avenae*.

The synthetic wheat lines ‘98-10-30’ and ‘98-10-35’ are the offspring of *T*. *aestivum* (var. Chris) and *T*. *turgidum* (var. durum) hybridization. Both lines have high hydroxamic acid, DIMBOA (2, 4-dihydroxy-7-methoxy-1, 4-benzoxazin-3-one) content [26], and appeared to be resistant to *S*. *avenae* under laboratory conditions [27]. In our study, we detected fewer aphids on ‘98-10-30’ in the field; yet, yield losses were high. Our results support those obtained by Li et al. [36]. The resistance mechanisms of ‘98-10-30’ are antibiosis and no tolerance. ‘98-10-35’ exhibited highly variable IAS and yield loss rates among different fields and years. In contrast, our results differed to those reporting lower IAS on ‘98-10-35’ in one year [80]. Highly variable IAS, KW, TY, and AY yield loss rates were obtained for the hybrid offspring of *T*. *aestivum* and wheat related species, ‘Tam200,’ ‘Tp,’ ‘Xiaoyan22,’ ‘PI high,’ and ‘98-10-35, even among 3 repeats in 2005.’ Thus, we conjecture that the quantitative trait locus (QTL) influences resistance in heterozygote and control varieties/lines, in addition to variation in meteorological conditions.

Our results identified ‘Tm’ (*Triticum monococcum*) as a resistant germplasm in all repeats over the 3 years. The resistance mechanism of this variety is antibiosis and non-preference. Seventy percent of *S*. *avenae* alatae preferentially fed on ‘Xinong1376’ after 24 h when given the choice between 2 wheat varieties. ‘Xinong1376’ contained large numbers of aphids, and had high yield loss rates in most repeats. This result was similar to that obtained by Li et al. and Liu et al. [35, 37]. ‘Xinong1376’ is an early maturing wheat cultivar that is used as a susceptible control variety to *S*. *avenae* in China [25, 26, 37, 77, 80, 81]. The development time of *S*. *avenae* on‘Xinong1376’ was shorter compared to other varieties, with greater fecundity and intrinsic rate of increase than other varieties [77].

We found that 67.8% of *S*. *avenae* alatae preferentially fed on ‘Xiyan22’ after 24 h when given the choice between 2 wheat varieties. However, in the field the average number of aphids found on this variety greatly varied across the 3 years. Since the end of the 20^th^ century, ‘Xiyan22’ has been a leading wheat cultivar in central Shaanxi Province. *S*. *avenae* must pierce more cell layers in the mesophyll of ‘Xiaoyan22’ compared to ‘Ww2730’ and ‘Batis’ [79]. Consequently, the aphid must salivate more before it can passively ingest the sieve elements of ‘Xiaoyan22’ compared to ‘Xinong 979’ and ‘Aikang58’ [82]. This difference leads to *S*. *avenae* that develop on ‘Xiaoyan22’ having a longer development time [77, 79], lighter body weight, and less offspring than those that develop on ‘Xinong 979’ and ‘Aikang58’ [82]. However, after 9 days feeding by *S*. *avenae*, ‘Xinong979’ and ‘Aikang58’ had lower fresh and dry matter loss rates than ‘Xiaoyan22’ [82]. This result indicates that ‘Xiaoyan22’ has excellent tolerance to *S*. *avenae*.

We detected high variation in the IAS and yield loss rate variations of ‘186Tm,’ ‘Tp,’ ‘Tam200,’ and ‘PI high’ in different fields and years, similar to that detected for ‘90-10-35.’ The resistance of some wheat varieties to *S*. *avenae* may be controlled by incomplete dominant genes [24] or dominant major genes [36, 80, 83–85]. However, for most intermediate or low resistance wheat varieties, resistance to *S*. *avenae* is regulated by a quantitative trait locus (QTL), which, in turn, is controlled the by micro effect of multiple genes, with resistance levels being influenced by environmental factors and aphid adaptability.

Our results showed that the yield loss rate of some wheat varieties/lines broadly varied across years. However, such variation was less for ‘Amigo,’ ‘Xiaoyan22,’ and some sample points of ‘186Tm’ compared to other varieties subject to similar or higher aphid numbers (Fig 6). In general, wheat yield loss is not noticeable at 4 aphids/tiller, but rapidly increases from 4 to 10 aphids/tiller in flowering and grain-filling period. However, in our study, 15 to 40 aphids/tiller caused less than 10% damage when compared to controls (without aphids) in some year. Thus, yield loss rate is relative to aphid number, damage duration time, and damage period [86]. Our results show that the extent of damage varies across years and with respect to the inherited characteristics of different wheat varieties/lines.

Screening for resistant germplasm in the laboratory may produce different results to field screening. Field screening is used to determine the resistance level of wheat germplasm to aphids based on natural aphid numbers that occur on wheat varieties [2]. This technique closely reflects natural conditions, and is suitable for the preliminary screening of large quantities of wheat germplasm materials. Resistance index score units are 0.25 in some references [36, 76] and 0.3 in others [25, 34, 35, 39]. Some studies have investigated the IAS in relation to the flowering and filling stage [30, 39, 42, 87], while others have investigated population dynamics throughout the entire wheat growth period [3, 38, 88, 89]. The MAN and ACC of aphid populations may differ due to different climatic conditions in different years, coupled with changes in the aggregation distribution of aphids in fields. Consequently, to guarantee that the sample error is not too large, the sample size should be large enough for the wheat field being tested [58, 59]. For instance, in our study, the repeat block area used for planting each wheat variety was 20 m^2^, with the sample area for the 5-point sampling being 0.25 m^2^, in which 80–130 wheat plants were planted.

In conclusion, our study demonstrates that the detected resistance of wheat varieties to *S*. *avenae* aphids may differ between laboratory and field experiments, due to the different settings. In addition, under field conditions, the resistance of different varieties differs across fields and years due to the way in which aphids aggregate, as well as differences in the genetic mixing of aphid stocks across years and climatic conditions. Therefore, we strongly recommend a combination of laboratory and long-term field experiments in the target planting regions to identify varieties/lines that consistently show high resistance to *S*. *avenae* infestation. Our study suggests that ‘Amigo’, ‘Astron,’ ‘Xanthus,’ and ‘Batis’ use antibiosis as the resistant mechanism. ‘98-10-30,’ ‘Astron,’ ‘Xanthus,’ ‘Ww2730,’ and ‘Tm’ also use antibiosis. ‘Amigo,’ ‘Xiaoyan22,’ and some ‘186Tm’ samples had good tolerance. Aphid population size and wheat yield loss rates greatly varied in different fields and years for ‘98-10-35,’ ‘Tp,’ ‘Tam200,’ ‘PI high,’ and other ‘186Tm’ samples, all these germplasm should be considered for use in future studies.

## Supporting Information

S1 Table Resistant germplasm in China. (DOCX)

## References

[1] Thackray DJ, Diggle AJ, Jones RAC. BYDV predictor: a simulation model to predict aphid arrival, epidemics of Barley yellow dwarf virus and yield losses in wheat crops in a Mediterranean-type environment. Plant Pathol. 2009; 58: 186–202.

[2] Liu X-F, Hu X-S, Keller MA, Zhao H-Y, Wu Y-F, Liu T-X. Tripartite interactions of barley yellow dwarf virus, *Sitobion avenae* and wheat varieties. PLoS ONE. 2014; 9(9): e106639 10.1371/journal.pone.0106639 25184214 PMC4153664

[3] Hu X-S, Zhao H-Y, Heimbach U, Thieme T, Li J, Zhang Y-H, et al Study on cereal aphid resistance on three winter wheat cultivars introduced into China. Acta Bota Bor-Occid Sin. 2004; 24: 1221–1226.

[4] Peng L-N, Zhang X-P, Ye J-S, Zuo Y. Study of resistance to insecticides in *Macrosiphum avenae* F. in Sichuan province. Chin J Pesticide Sci. 2000; 3: 13–18.

[5] Sun J-W, Wang J-L, Li S-J, Wang X-Y. The effect of wheat varieties on the population dynamic of wheat aphid. J Henan Agr Sci. 1993; 1: 26–28.

[6] Lowe HJB. Characteristics of resistance to the grain aphid *Sitobion avenae* in field trials. J Agri Sci. 1984; 102: 487–497.

[7] Havličková H. Level and nature of the resistance to the cereal Aphid, *Sitobion avenae* (F.), in thirteen winter wheat cultivars. J Agro Crop Sci. 1993; 171(2): 133–137.

[8] Riazuddin MA, Khattak K. Screening resistant wheat lines against aphids. Pak Entomol. 2004; 26: 13–18.

[9] Caillaud CM, Dedryver CA, DiPietro JP, Simon JC, Fima F, Chaubet B. Clonal variability in the response of *Sitobion avenae* (Homoptera: Aphididae) to resistant and susceptible wheat. Bull Entomol Res. 1995; 85(2): 189–195.

[10] Di Pietro JP, Caillaud CM, Chaubet B, Pierre JS, Trottet M. Variation in resistance to the grain aphid, *Sitobion avenae* (Sternorhynca: Aphididae), among diploid wheat genotypes: Multivariate analysis of agronomic data. Plant Breeding. 1998; 117(5): 407–412.

[11] Silva AM, Sampaio MV, de Oliveira RS, Korndorfer AP, Ferreira SE, Polastro GC, et al Antibiosis and non-preference of *Sitobion avenae* (F.) (Hemiptera: Aphididae) on leaves and ears of commercial cultivars of wheat (*Triticum aestivum*). Neotrop Entomol. 2013; 42(3): 304–310. 10.1007/s13744-013-0117-5 23949814

[12] Dogimont C, Bendahmane A, Chovelon V, Boissot N. Host plant resistance to aphids in cultivated crops: genetic and molecular bases, and interactions with aphid populations. C R Biol. 2010; 333: 566–573. 10.1016/j.crvi.2010.04.003 20541167

[13] Hesler LS, Tharp CI. Antibiosis and antixenosis to *Rhopalosiphum padi* among triticale accessions. Euphytica. 2005; 143: 153–160.

[14] Cailaud CM, Dedryver CA, Simon JC. Development and reproductive potential of the cereal aphid *Sitobion avenae* on resistant wheat lines (*Triticum monococcum*). Ann Appl Biol. 1994; 125: 219–232.

[15] Liu Y, Ni H-X, Sun J-R, Hu C. The effects of wheat varieties resistant to aphids on the pupolation of *Sitobion avenae* (Fabricius) and parasitization as well as development of its parasitic wasp *Aphidius avenae* haliday. Acta Phytophyl Sin. 2001; 28: 203–206.

[16] Cai Q-N, Ma X-M, Zhao X, Cao Y-Z, Yang X-Q. Effects of host plant resistance on insect pests and its parasitoid: A case study of wheat-aphid–parasitoid system. Biol Control, 2009; 49: 134–138.

[17] Smith MC, Quisenberry SS, DuToit F. The value conserved wheat germplasm evaluated for arthropod resistance In: Clement SC, Quisenberry SS, eds. Global genetic plant resources for insect-resistant crops. Boca Raton Boston London New York Washington DC CRC Press 1999; 25–49.

[18] Hollenhorst MM, Joppa LR. Chromosomal location of genes for resistance to greenbug in ‘Largo’ and ‘Amigo’ wheats. Crop Sci. 1983; 1: 91–93.

[19] Graybosch RA, Lee JH, Peterson CJ. Genetic, agronomic and quality comparisons of two 1AL. 1RS. wheat-rye chromosomal translocations. Plant Breeding. 1999; 2: 125–130.

[20] Zheng W-Y, Yin Q-Y, Shi Z-L, Ma A-P. Review of the resistant mechanism and genetic of winter wheat varieties to *Sitobion avenae*. J Wheat Research. 1999; 1:, 1–5.

[21] Ding H-S. The investigation of wheat aphid in Guizhou province. Entomol Knowle. 1958; 2: 70–74.

[22] Zhu H-F, Han Y-F, Wang L-Y. The populations of wheat insects under different cultural conditions of wheat. Acta Entomol Sin. 1961; 4–6: 411–424.

[23] Wu R-J, Yang J-G, Gao Y-Y. Bioassay on tolerance of different wheat varieties to wheat aphids. Plant Prot Tech Extension. 1996; l: 3–5.

[24] Yin Q-Y, Zheng W-Y, Liu L-L. The progress of resistant wheat germplasm and their resistant mechanism. J Wheat Research. 2003; 3: 30–36.

[25] Li S-J, Zhang Z-Y, Wang X-Y, Ding H-J, Ni H-X, Sun J-R, et al Identifying wheat species (lines) by fuzzy recognition method. Plant Prot. 1998; 5: 15–16.

[26] Li S-J, Liu A-Z, Wu Y-Q, Li S-G, Lu X-H, Yin H-E. Population dynamics of wheat aphids and natural enemies in different wheat varieties. Entomol Knowle. 2001; 38: 355–358.

[27] Du L-F, Zhao H-Y, Yuan F, Sun Q, Zhang G-S, Yao J-X, et al Resistance to aphid determining and screening in wheat species (lines) or sources. Acta Boreal-Occident Sin. 1999; 19: 68–73.

[28] Hu X-S, Liu X-F, Hu Z-Q, Zhang Y-H, Zhao H-Y, Zhang G-S. The resistance of 10 wheat varieties to *Sitobion avenae* (Homoptera: Aphididae) in wheat seedlings phase in lab. Plant Prot. 2011; 37: 81–85.

[29] Hu X-S, Keller MA, Liu X-F, Hu Z-Q, Zhang Y-H, Zhao H-Y, et al The resistance and correlation analysis to three species of cereal aphids (Hemiptera: Aphididae) on 10 wheat varieties orlines. J Econ Entomol. 2013; 106: 1894–1901. 24020308 10.1603/ec13071

[30] Qu H-X, Dang J-Y, Cheng M-F, Xie X-S. Resistance identification of wheat germpiasm resources to *Macrosiphum auenae*. Acta Agr Boreali-Sin. 2004; 4: 102–104.

[31] Yu Y, Pang B-P, Gao S-Y, Xia C-Y. Effects of spring wheat varieties on growth, development and fecundity of *Sitobion avenae*(F.) (Homoptera:Aphididae). Chin J Appl Ecol. 2006; 2: 354–356. 16706070

[32] Li X-Q, Guo X-R, Li K-B, Yin J, Cao Y-Z. Resistance of wheat varieties (lines) to *Sitobion miscanthi* (Takahashi) (Aphidoidea:Aphididae). Acta Entomol Sin. 2006; 49: 963–968.

[33] Dong H. The research of resistant wheat varieties (lines) to aphid and their resistant mechanism. Dissertation for master degree, Shandong Agriculture University. Taian, Shandong. 2006.

[34] Liu X-L, Wang C-Y, Wang Y-J, Sang L-Q, Xiang J-Y, Ji W-Q. Relationship between morphological characters of wheat germplasm and their resistance to *Sitobion avenae* (F). J Triticeae Crops. 2006; 6: 24–28.

[35] Liu X-L, Wang C-Y, Wang Y-J, Zhang H, Ji W-Q. Screening and evaluation of different wheat varieties for resistance to English grain aphid *Sitobion avenae* at seedling and adult-plant stages. Acta Phytophyl Sin. 2014; 41(2): 216–224.

[36] Duan C-X, Wang X-M, Zhu Z-D. Screening and evaluation of wheat germplasm for resistance to the aphid (Sitobion avenae). J Plant Genetic Resources. 2006; 3: 297–300.

[37] Li J, Zhao H-Y, Li Z-G, Han S-C, An X-C. Resistance of different wheat varieties to *Maerosiphum avenae*. Chin Bull Entomol. 2007; 44: 509–512.

[38] Wang M-F, Yang H-M, Liu J-Q, Lei Z-S, Wu Z-Q, Yuan G-H, et al Effect of aphid damage on wheat yield and quality in Yellow and Huai Valleys winter wheat region. J Hennan Agr Sci. 2011; 4: 16–20.

[39] Qu F, Dang J-Y, Cheng M-F, Lian J, Xie X-S. Resistance identification of new variety wheat to *Macrosiphum avenae*. J. Shanxi Agr Sci. 2012; 4: 386–388, 392.

[40] Li F-Q, Kong L-R, Liu Y-S, Wang H-Z, Peng J-H. TOPSIS based comprehensive evaluation of the resistance in wheat germplasm to English grain aphid. Plant Sci J. 2013; 3: 228–241.

[41] Li F-Q, Kong L-R, Liu Y-S, Wang H-Z, Chen L, Peng J-H. Response of wheat germplasm to infestation of English grain aphid (Hemiptera: Aphididae). J Econ Entomol. 2013; 106: 1473–1478. 23865216 10.1603/ec12327

[42] Lu Z-Y, Gao Z-L, Dang Z-H, Li Y-F, Li J-C, Liu W-X, et al Identify and evaluation of resistance of wheat varieties to aphids. J Hebei Agr Sci. 2014; 3: 24–26.

[43] Xu L-J, Lv Y-H, Duan X-L, Zhang X-H, Liang R-Q. Evaluation of wheat cultivars/lines for resistance to aphids at booting and filling stage. J China Agr University. 2014; 1: 21–28.

[44] Xie N, Wang L, Qi S-S, Liu W-X, Wu Y-Q. Resistance analysis of wheat relatives to *Sitobion avenae*. Henan Sci. 2014; 3: 1020–1023.

[45] Liang H, Zhu Y-F, Zhu Z, Sun D-F, Jia X. Obtainment of transgenic wheat with the insecticidal Lectin from snowdrop (*Galanthus nivalis* agglutinin; GNA) gene and analysis of resistance to aphid. Acta Genet Sin. 2004; 2: 189–194. 15473311

[46] Xu Q-F, Tian F, Chen X, Li L-C, Lin Z-S, Mo Y, et al Molecular test and aphids resistance identification of new transgenic wheat lines with GNA gene. J Triticeae Crops. 2005; 3: 7–10.

[47] Yu Y, Wei Z-M. Increased oriental armyworm and aphid resistance in transgenic wheat stably expressing *Bacillus thuringiensis* (Bt) endotoxin and *Pinellia ternate* agglutinin (PTA). Plant Cell Tiss Organ Ctm. 2008; 94: 33–44.

[48] Pan J-X. Identification and screening of new transgenic wheat lines of wheat aphid resistance. Hebei University Sci Tech. Dissertation for the Master Degree, Shijiazhaung. 2013.

[49] Ou X-Q, Ru Z-G, Hu T-Z, Shi M-W. Tolerance of the main wheat cultivars in Henan province to wheat aphids. J Triticeae Crops. 2005; 2: 125–127.

[50] Painter R. Insect resistance in crop plants. MacMillan Press, New York, NY 1951.

[51] Panda N, Khush SG. Host plant resistance to insects. CABI, Wallingford, UK 1995; 448.

[52] Liu S-Y, Hou Y-M, Li D-X, Gao C-S. The resistance on the resistive mechanism of wheat varieties to English grain aphid *Maerosiphum avenae* (F.) Acta Agr Boreali-Occident Sin. 1993; 3: 76–80.

[53] Wang Y-M, Zhao Z-J. The long-distance migration regularity and cereal virus control strategy in North of Shanxi province. J Shanxi Agr Sci. 1983; 04: 16–19.

[54] Zhang X-C, Zhou G-H, Shi M, Fang J-Z, Zhao Z-P, Li S-H, et al Study on the long-distance migration of and virus transmission by the aphid *Sitobion averae*(F.). Acta Phytophyl Sin. 1985; 1: 9–15.

[55] Wei H-T, Li J, Wu C, Xiang Y-J, Li C-S, Yang W-Y, et al Resistance of major wheat varieties to *Rhopalosiphum padi* (Linnaeus) in Sichuan Province. Chin J Appl Entom. 2014; 51(6): 1524–1531.

[56] Hu X-S, Zhao H-Y. The reviews of wheat resistant mechanism to cereal aphid in China, Chin J Appl Entomo. 2014; 51(6): 1459–146.

[57] Xu L-J, Lv Y-H, Duan X-L, Zhang X-H, Liang R-Q. Evaluation of wheat cultivars/lines for resistance to aphid at booting and filling stages. J China Agr University. 2014; 19(1): 21–28.

[58] Qi M-W, Zhang D-C. A study on the spatial distribution and sampling method of wheat aphids. J Shandong Agr University. 1987; 4: 59–64.

[59] Zhang R-L, Xu Y-X, Yu S-Y. A study on the spatial distribution and sampling method of *Sitobion avenae*. Entomol Knowle. 1999; 3: 135–137.

[60] Boeve PJ, Weiss M. Spatial distribution and sampling plans with fixed levels of precision for cereal aphids (Homoptera: Aphididae) infesting spring wheat. Can Entomol. 1998; 130: 67–77.

[61] Fievet V, Dedryver C-A, Plantegenest M, Simon J-C, Outreman Y. Aphid colony turn-over influences the spatial distribution of the grain aphid *Sitobion avenae* over the wheat growing season. Agr Forest Entomol. 2007; 9: 125–134.

[62] Winder L, Perry JN, Holland JM. The spatial and temporal distribution of the grain aphid *Sitobion avenae* in winter wheat. Entomol Exp Appl. 1999; 93: 277–290.62.

[63] Llewellyn KS, Loxdale HD, Harrington R, Brookes CP, Clark SJ, Sunnucks P. Migration and genetic structure of the grain aphid (*Sitobion avenae*) in Britain related to climate and clonal fluctuation as revealed using microsatellites. Mol Ecol. 2003; 12 (1): 21–34. 12492875 10.1046/j.1365-294x.2003.01703.x

[64] Llewellyn KS, Loxdale HD, Harrington R, Clark SJ, Sunnucks P. Evidence for gene flow and local clonal selection in field populations of the grain aphid (*Sitobion avenae*) in Britain revealed using microsatellites. Heredity. 2004; 93(2): 143–153. 15241466 10.1038/sj.hdy.6800466

[65] Simon JC, Baumann S, Sunnucks SP, Herbert PDN, Pierre JS, Le Gallic J-F, et al Reproductive mode and population genetic structure of the cereal aphid Sitobion avenae studied using phenotypic and microsatellite markers. Mol Ecol. 1999; 8: 531–545. 10327655 10.1046/j.1365-294x.1999.00583.x

[66] Jensen AB, Hansen LM, Eilenberg J. Grain aphid population structure: no effect of fungal infections in a 2-year field study in Denmark. Agr and Forest Entomol. 2008; 10(3): 279–290.

[67] Papura D, Simon J-C, Halkett F, Delmotte F, Le Gallic J-F, Dedryver C-A. Predominance of sexual reproduction in Romanian populations of the aphid *Sitobion avenae* inferred from phenotypic and genetic structure. Heredity. 2003; 90, 397–404. 12714986 10.1038/sj.hdy.6800262

[68] Figueroa CC, Simon JC, Le Gallic JF, Prunier-Leterme N, Briones LM, Dedryver C-A, et al Genetic structure and clonal diversity of and introduced pest in Chile, the cereal aphid *Sitobion avenae*. Heredity. 2005; 95: 24–33. 15931255 10.1038/sj.hdy.6800662

[69] Cai F-H, Zhao H-Y. Genetic polymorphism in natural populations of *Macrosiphum avenae*. J Northwest A&F University (Natu ScI Ed). 2004; 32 (2): 21–24.69.

[70] Xu Z-H, Chen J-L, Cheng D-F, Sun J-R, Liu Y, Francis F. Discovery of English grain aphid (Hemiptera: Aphididae) biotypes in China. J Econ Entomol. 2011; 104(3): 1080–1086.70. 21735932 10.1603/ec10204

[71] Xin J-J, Shang Q-L, Desneux N, Gao X-W. Genetic diversity of *Sitobion avenae* (Homoptera: Aphididae) populations from different geographic gegions in China. PLoS ONE. 2014; 9(10): e109349 10.1371/journal.pone.010934971 25356548 PMC4214629

[72] Huang X-L, Liu D-G, Wang D, Shi X-Q, Simon J-C. Molecular and quantitative genetic differentiation in *Sitobion avenae* populations from both sides of the Qinling mountains. PLoS ONE. 2015; 10(3): e0122343 10.1371/journal.pone.0122343 25822721 PMC4379161

[73] Dong Q-Z, Wei K, Meng Q-X, Wu F-Z, Zhang G-X, Zhong T-S, et al Investigation on long distance migration of grain aphid (*Macrosiphum avenae* (Fabr.)) in NingXia. Acta Entomol Sin. 1987; 3: 277–284.

[74] Luo R-W, Yang C-L, Shang Y-F, Li C-S. Study on the sourece of English grain aphid. Acta Phytophyl Sin. 1988; 3: 153–158.

[75] Zhao Z-J. The overwintering sites of wheat aphids in Wuwei. Plant Prot. 1996; 2: 78.

[76] Ye S-H, Chen A-J, Shi G-Y. Roles of spring wheat physical features in the resistance to *Sitobion avenae* F. Grassland and Turf. 2012; 3: 78–80, 83.

[77] Wang C-P, Luo K, Zhu Q-D, Li D, Zhao H-Y, Zhang G-S. Analysis on resistance of wheat germplasm by GIS to *Sitobion avenae*. J Northwest A & F University (Natu Sci Ed). 2011; 4: 41–47.

[78] Thieme T, Heimbach U. Development and reproductive of cereal aphids (Homoptera: Aphididae) on winter wheat cultivals. Wprs Bull. 1996; 19: 1–8.

[79] Hu X-S, Zhao H-Y, Hu Z-Q, Li D-H, Zhang Y-H. EPG Comparison of *Sitobion avenae* (Fab.) feeding behavior on three wheat varieties. Agr Sci China. 2008; 2: 180–186.

[80] Wang C-P, Wang Z-H, Zhao H-Y, Zhu Q-D, Luo K, Wang LM, et al Expression of potential resistance genes to the English grain aphid, *Sitobion avenae*, in wheat, *Triticum aestivum*. J Insect Sci. 2013; 13: 90 10.1673/031.013.9001 24205793 PMC3835026

[81] Zhang Z-Y, Li S-J, Zhang Y-D, Wang X-Y, Li S-G. Study on winter wheat resistance to aphids in different developmental stage. Acta Agr Boreali-Sin. 2000; 1: 57–61.

[82] Cao H-H. Constitutive and induced defences of wheat against *Sitobion avenae*. Dissertation for doctor degree Northwest A&F University, Yangling, China. 2014.

[83] Liu X-L, Yang X-F, Wang C-Y, Wang Y-J, Zhang H, Ji W-Q. Molecular mapping of resistance gene to English grain aphid (*Sitobion avenae* F.) in *Triticum durum* wheat line C273. Theo Appl Genet. 2012; 124: 287–293. 10.1007/s00122-011-1704-7 21953208

[84] Hu B-F, Ma X-L, Shi G-Y, Li B-C, Meng Y-X, Shang X-W, et al Genetic analysis of aphid (*Macrosiphum avenae*) resistant in spring wheat line‘J-31’and‘J-48’. J Gansu Agr University. 2009; 3: 58–63.

[85] Fu J, Zhang S-H, Wen S-M, Yang X-J. SSR markers linked to a wheat aphis resistance gene. J Agr University of Hebei, 2008; 31(5): 1–4.

[86] Larsson H. A crop loss model and economic thresholds for the grain aphid, *Sitobion avenae* (F.), in winter wheat in southern Sweden. Crop Prot. 2005; 24: 397–405.

[87] Guo X, Li K-B, Yin J, Wang B, Cao Y-Z. Effects of wheat varieties on population parameters of *Macrosiphum avenae* (Fabricius). Sci Agr Sin. 2010; 43: 2056–2063.

[88] Wu W-Q, Piyaratne MKDK, Zhao H-Y, Li C-L, Hu Z-Q, Hu X-S. Butterfly catastrophe model for wheat aphid population dynamics: Construction, analysis and application. Ecol Mode. 2014; 88: 55–61.

[89] Shi Z-L, Zheng W-Y, Yi Q-Y, Ma A-P, Xu G-Y. A study on field selecting technique of wheat varieties for resistance to wheat aphid. Acta Agric Boreali-Sin. 1999; 14(1): 98–101.

