# Supplementary material for: Resistance of Wheat Accessions to the English Grain Aphid Sitobion avenae
Source: PLoS One. 2016 Jun 1;11(6):e0156158. doi: 10.1371/journal.pone.0156158 (PMC4889116; doi:10.1371/journal.pone.0156158)
Supplement: S1 Table — (DOCX) [file pone.0156158.s001.docx]

| Resistant gelmplasm | screening and identifying method | references |
| --- | --- | --- |
| Hr: Nongda4356, Yanda1817, Nongda198; R: Nongda6085, Mangbai4-2 |  | Zhou et al 1982 |
| Hr: Linyuan207, R: Weidong8, Yan7578-128, 77(4)-10 | Three year, average aphid number, field | Zheng et al 1987 |
| Hr: Shan167, 88(37)65; Mr: 7212, 82(34)0-7, (87)113, 82246; Lr: 81168, Ji21, 8121, 80-1, Shan213. | Life-table, field and lab, natural infestation at adult plant stage in field, 1 year | Gao 1994, 1995 |
| Linyuan28013, Linkang1 |  | Yin et al 1998 |
| *Triticum monococcum*, 98-10-9 | Natural infestation at adult plant stage in field; multiobjectives decision-making and optimization | Du et al 1999 |
| Hr: Zhong4 no awn, KOK(1679), Mr: Lovering10, beijing10, Chul(1497) , L1, JP①; Lr: Xiaobaidongmai, Xinong6028, Fenchan3 | Natural infestation at adult plant stage in fields; aphid mode number, IAS | Li et al 1998, 2001 |
| 84(G) 6; Xiaoyan107; 881-16-9; Xinong34- 9; 8222; JInmai27 | Manual inoculation in lab | Wu et al 1995, 1999 |
| Hr: Jinying117, Jinying158, Shandong924402-6, Red awn (Hongmanghong), JPD, ZM23774, MY295, nongda3214, Zhong4 no awn,95zhong44, Canghe030; Mr(63 wheat varieties); Lr(110wheat varietes); Stable resistance (32 wheat varieties); Resistant to S. avenae, rust and Powdery mildew: KM75.4552, TE4732A, RAPID, ROUFA, CIT920080, ARIESAN | Natural infestation at adult plant stage in 4 fields; aphid peak number, IAS | Qu et al 2004 |
| Hr: Zhong4no awn, JP①; Mr: JiBao1 | IAS, Lab | Li et al 2006 |
| Mr: Kanghan61, 919, 4443, 981713; R: 009, 4855, 85, 8086, 6065 | Natural infestation at adult plant stage in field; average aphid number, IAS,1 years | Dong 2006 |
| Hr: C273,Lanmai(Shaanxi Zashui); Mr: Yumai, Huhansan, Laomai, YumaiS1125, YumaiS1111,YumaiS1124, Jana; Lr(21 wheat varieties) | Natural infestation at adult plant stage in field; Average aphid number, IAS, 3 years | Liu et al 2006 |
| Hr:C273,Lanmai(Shaanxi Zashui); Mr: PI137739, PI262260, PI294994, Hongmanghong(Red awn, Yu324), Liying6, Xiaobaidongmai, Yanda1817, Zhong4 no awn, Yumai(Xunyang1), Yumai(Xunyang2), Yumai(Xunyang3), bailanmai(Xunyang), Yumai(Baihe), Huhanshan(Shanyang), mazamai, PI294994 | Natural infestation at adult plant stage in field; Manual inoculation on seedling in greenhouse, 3 years | Liu et al 2014 |
| Hr: TM44, XM800, Linyuan207,XM740,MY3561,PI372129; R: Sigeassons, KOK1679, Zhongpin1818, MY8011, PI137739, Zhongpin1817, Xiaomai, Xuzhou15), Mr:F1 of Shannong7859×KOK1679, Liying5, MY295/Atlas66, PI243781, Stphens, Jiyu1051, ZM10216, PI262660, Clark, Diaodiaomai, Baimaizi, ZM10215, Liying6, MY5869, MY4989, PI294994, Mianyang72-34, Luohan1, Zhongda89-8092, Lovrin36. | Natural infestation at adult plant stage in field; Manual inoculation on seedling in greenhouse, Aphid peak number, IAS, 4 years | Duan et al 2006 |
| Ww2730, Astron, 98-10-30, 98-10-35 | Manual inoculation at seedling stage in lab | Hu et al 2011 |
| Hr: haocheng8901, ZM23774, ShiL4058, Linxuan6214, Yumai68, Shanyou225, Lankao greater ear, Yumai47, Jimai26, Xinmai19, Xu4043, Yannong21, Shannong1730, Huimai0209, Henong1150, Linxuan2035, 96(60)/8-3-2, 99(66)/8-3-2, Annong0487, Shannong14, ShiL4021, Fenyou7, Linxuan2044, Han7086, B99360-1-2, Xu5034, Xu4060; MR: 93 wheat varieties, Lr: 123 varieties. | Natural infestation, at adult plant stage in field, 3 years | Qu et al 2012 |
| Zhengkekang, Jimai418, Henong825, ShiB07-4056, Hen4338, Cangmai119, Jiemai-19, Hen5229, Xinmai6, Cangmai2009-24, Gaoyou9618, Han4589, Gaoyou9908, Bao39. | Natural infestation at adult plant stage in field, 1 year | Lu et al 2014 |
| Hr: hen6632, nongda3432, JIngdong8, R: Luomai4, Han6228, Han4015, KOK1679, Yannong23, Jinhua1, Linkang15, Yangmai158; Mr: 08P20, KOK1679, Hen6632, Ji38, Linkang15, Luomai4 | Natural infestation at adult plant stage in field, 2 years | Xu et al 2014 |
| Hr: *Psathyrostachys juncea*(Qinghe), *Roegneria karelinii*(Mulei), *Elytrigia repens*(Buerjin), *Leymus angustus* | Manual inoculation in field | Ma 1994 |
| Hr: Aegilops kotschyi var．variabilis)(No.AS26, AS28), Ae．biuncialis(No. AS48), Ae．Juvenalis(No.ASll2) | Natural infestation at adult plant stage in field, 1 year | Chen 2005 |
| Aegilops geniculata chromosome addition line with high resistance, the resistance genes come from the chromosomes of Aegilops geniculata’s 1M^g^ and 7M^g^ . | Natural infestation at adult plant stage in field, 1 year | Xie et al 2014 |
| Hr: PI623081 (Iran); Mr: Guan0537, Silver in gold (Jinbaoying), PI294994, PI220217, Wanmai18, Emai6, Huamai8号, WE27, Han6172, Pollen culture5(Huapei5), Chuanmai107, JImai6, Qianmai15, Guinong13, Guimai5, Guifen4, PI415154, Cltr17882, PI134770; Lr(35 wheat varieties) | Natural infestation at adult plant stage in field; Average aphid number, IAS, 3 years | Li et al 2013 |
| Hr: Shan253, Yumai70, Shanru1, 186tm; Mr (55 wheat varieties, including Amigo, 98-10-35, Xiaoyan22, Pi High, Changwu612, Kaifen18, Sumai3, Shan715, 3399, CBJ10, Zhi2155, BBJ4, Zhoumai19, Zhouyou102, Mei8, LunXuan069), Lr(113 wheat varieties) | Natural infestation at adult plant stage in field; Aphids manual inoculation at filling stage in field, 3 years | Wang et al 2011 |
| Hr: C272,C151,04-9284; R: 04-9471,04-9472,04-9547, 04-9561,C141,CZs6,04-9333,04-9225,Guinong21 | Manual inoculation at filling stage in field, average aphid number (IAS), 3 years, Spring wheat | Shi et al 2008 |
| Hr: 04-9225, 04-9284; R: 04-9283, C141, C151, 04-9474, C152, C272, 04-9333, 04-9282, Guinong21; Lr(42 wheat varieties) | Average aphid number, field, Spring wheat, 3 years | Ye et al 2012 |
| Hr: Pm232, Jibao1, FH640; Mr: Zhong1506, 781G, Yan7578; Lr: Aizao781, Taian2507, Aifen3 | Manual inoculation | Guo et al 2010 |
| Tolerance: Tm44, AmigoF3, Amigo, AmigoF2, AmigoF4, 98-10-9; Antibiotic: Largo, 98-10-32, Shan229, 98-10-35, XZl3mother, PI high, XZl 3rd generation | Cumulative survival rate, Yeild loss rate | Li et al 2007 |
| Hr: KOK1679, ChuI1497; Mr: Zhengzhou831, Yanda872 | Field, 3 years | Sun et al 1993 |
| Tolerance: Jingdong6, beinong2, Jingshuai16; nongda91, JIng441 | Natural infestation at adult plant stage in field, 2 years | Wu et al 1996 |
| Yanda1817, Nongda7037, nongda4356, Mazamai(Shaanxi), baijianmai(Wudu) | Natural infestation at adult plant stage in field, fuzzy recognition method | Xia et al 1990 |

Note: Hr: high resistant; Mr middle resistant; Lr: low resistant.
